# Hyperammonemic coma after craniotomy: Hepatic encephalopathy from upper gastrointestinal hemorrhage or valproate side effect? Case report and literature review

**DOI:** 10.1097/MD.0000000000006588

**Published:** 2017-04-14

**Authors:** Xiaopeng Guo, Junji Wei, Lu Gao, Bing Xing, Zhiqin Xu

**Affiliations:** Department of Neurosurgery, Peking Union Medical College Hospital, Chinese Academy of Medical Sciences, Beijing, People's Republic of China.

**Keywords:** hepatic encephalopathy, postoperative coma, upper gastrointestinal hemorrhage, valproate, valproate-induced hyperammonemic encephalopathy

## Abstract

**Rationale:**

Postoperative coma is not uncommon in patients after craniotomy. It generally presents as mental state changes and is usually caused by intracranial hematoma, brain edema, or swelling. Hyperammonemia can also result in postoperative coma; however, it is rarely recognized as a potential cause in coma patients. Hyperammonemic coma is determined through a complicated differential diagnosis, and although it can also be induced as a side effect of valproate (VPA), this cause is frequently unrecognized or confused with upper gastrointestinal hemorrhage (UGH)-induced hepatic encephalopathy. We herein present a case of valproate-induced hyperammonemic encephalopathy (VHE) to illustrate the rarity of such cases and emphasize the importance of correct diagnosis and proper treatment.

**Patient concerns and diagnoses:**

A 61-year-old woman with meningioma was admitted into our hospital. Radical resection of the tumor was performed, and the patient recovered well as expected. After administration of valproate for 7 days, the patient was suddenly found in a deep coma, and her mental state deteriorated rapidly. The diagnoses of hepatic encephalopathy was confirmed. However, whether it origins from upper gastrointestinal hemorrhage or valproate side effect is uncertain.

**Interventions and outcomes:**

The patient's condition fluctuated without improvement during the subsequent 3 days under the treatment of reducing ammonia. With the discontinuation of valproate treatment, the patient regained complete consciousness within 48 hours, and her blood ammonia decreased to the normal range within 4 days.

**Lessons subsections:**

VHE is a rare but serious complication in patients after craniotomy and is diagnosed by mental state changes and elevated blood ammonia. Thus, the regular perioperative administration of VPA, which is frequently neglected as a cause of VHE, should be emphasized. In addition, excluding UGH prior to providing a diagnosis and immediately discontinuing VPA administration are recommended.

## 1 Introduction

Craniotomy has long been the first-line therapy for meningioma resection, and impaired consciousness is not uncommon after brain tumor resection.^[1,2]^ Brain swelling, intracranial hemorrhage, and cerebral infarction, which can be ruled out by a brain CT scan,^[3]^ are considered major causes of disturbances of consciousness.^[1,4]^ Valproate (VPA) is an effective and broad-spectrum antiepileptic drug (AED) that is mainly used in neurology and psychiatry, although it also acts as a routine medication in high-risk brain tumor patients to prevent postoperative seizures.^[3,5]^ VPA-induced hyperammonemic encephalopathy (VHE) is mostly asymptomatic, whereas the symptomatic disease is uncommon and fatal.^[6]^ The long-term or excessive consumption of VPA in the treatment of epilepsy is commonly reported as the cause of VHE.^[5]^ Because of the low dose, perioperative prophylactic administration of VPA rarely results in postoperative VHE, and it is often disregarded by neurosurgeons in clinical practice.

Herein, we present a case of a 61-year-old woman suffering from VHE after meningioma resection whose diagnosis was confused with postoperative upper gastrointestinal hemorrhage (UGH). Adjuvant therapy to reduce ammonia was not successful; however, after discontinuing VPA, the patient regained full consciousness within 48 hours. To the best of our knowledge, this is the first reported case of VHE after craniotomy with UGH, which is a complicated differential diagnosis. Because the rarity and disregard for VHE often leads to misdiagnoses and delayed treatment of fatal postoperative impaired consciousness,^[3]^ we also reviewed the literature in this field to provide recommendations for the proper differential diagnosis and timely management of such cases.

## 2 Case report

A 61-year-old Chinese woman complained of dizziness for 3 weeks and was admitted to our hospital after a bifrontal mass was found radiologically. Other than dizziness, the patient presented no signs or symptoms that are commonly associated with neurological disease, such as headache, vomiting, sensory disturbances, altered consciousness, or seizures. Except for a 20-year history of hypertension, which was properly controlled with angiotensin-converting enzyme inhibitors, the patient was otherwise healthy. No special circumstances were identified regarding her personal or family history, and the patient was found to be normal upon neurological examination.

**Figure 1:**
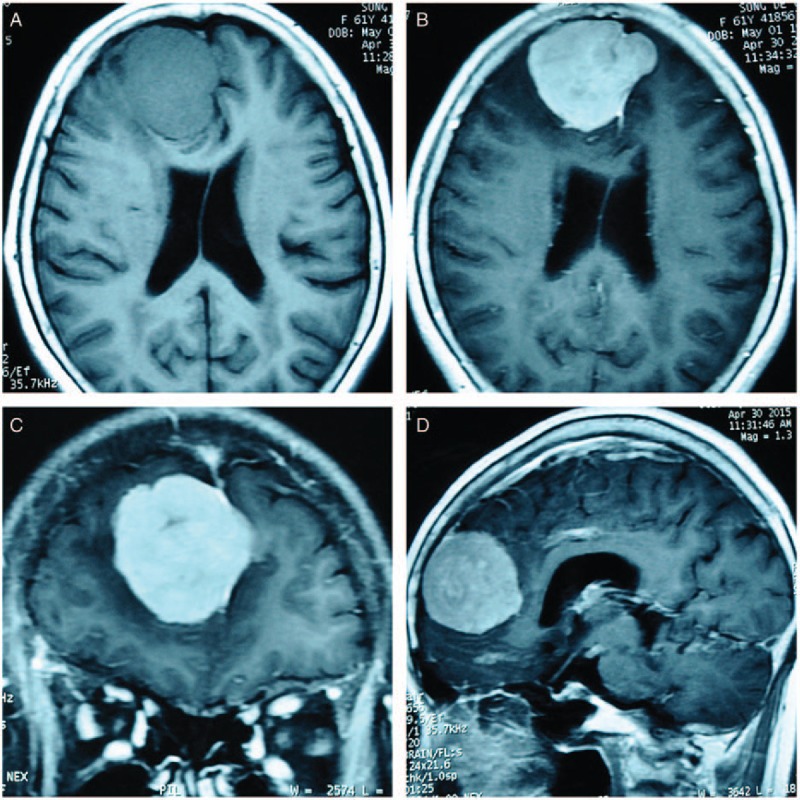
Preoperative plain and contrast-enhanced MRI of the head. The tumor is approximately 5.7 × 5.0 × 5.0 cm^3^ and has a characteristic “dural-tail” sign in the contrast-enhanced imaging. A is the axial view of the T1-weighted image, which shows an isointensity mass. The tumor is homogenously contrast-enhanced and shown in axial (B), coronal (C), and sagittal (D) views. MRI = magnetic resonance imaging.

Plain and contrast-enhanced magnetic resonance imaging (MRI) of the head showed a space-occupying lesion in the bifrontal lobe, mainly on the right side, clinging to the sagittal sinus and the cerebral falx. The 5.7 × 5.0 × 5.0 cm dural-based mass growing across the sagittal midline revealed isointensity on the T1-weighted image and a slight hyperintensity on the T2-weighted image and was homogenously enhanced with a characteristic “dural-tail” sign (Fig. 1). Diagnosis of a parasagittal-parafalcine meningioma was made, and a craniotomic meningioma resection was chosen as treatment.

The blood cell examination; coagulation function, liver and renal function tests; urinalysis; chest x-rays and electrocardiogram were normal. The albumin was 36 g/L, the total bilirubin (TBil) was 12.2 μmol/L, and the direct bilirubin (DBil) was 4.1 μmol/L. Alanine transaminase (ALT) was 17 U/L, and the international normalized ratio (INR) for coagulation values was 1.02. Because of the patient's advanced age and history of hypertension, we performed an echocardiograph and a blood gas analysis and tested her creatine kinase levels and pulmonary functions. The results revealed no surgical contraindications. Written consent was obtained from the patient.

Five hundred milligrams of VPA twice per day was initiated 3 days before the operation. The patient tolerated the administration well. A craniotomy was then performed with a radical tumor resection (Simpson II). The tumor was pink and solid and had a moderate blood supply, and its texture was medium. Thirty minutes before the end of surgery, 800 mg of VPA was administered by intravenous infusion. After the operation, the patient regained consciousness within 30 minutes and scored 15 on the Glasgow coma scale (GCS). Two hours later as she was being transported back to her ward, the patient underwent UGH. The vomitus (approximately 50 mL) was composed of coffee-colored blood. Excluding the possible etiology of endotracheal intubation damage, we diagnosed the patient as experiencing acute erosive-hemorrhagic gastritis, a reaction to the craniotomy injury. The patient was given omeprazole to inhibit gastric acid secretion, and the hematemesis ceased.

**Figure 2:**
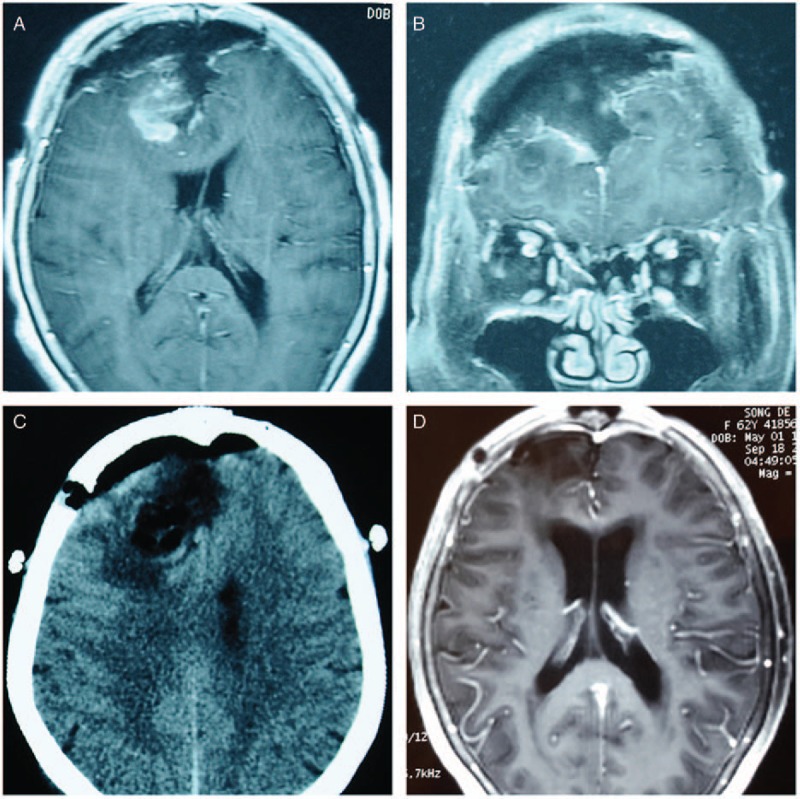
Radiological findings of the patient during the postoperative and follow-up periods. The axial (A) and coronal (B) views of the contrast-enhanced MRI on postoperative day 3 revealed the radical removal of the tumor and no sign of intracranial hemorrhage or brain swelling. Emergency brain CT (postoperative day 4) (C) failed to reveal any abnormal findings that would indicate brain swelling, intracranial hemorrhage, or cerebral infarction. A follow-up MRI 5 months postoperation (D) showed a clear operative field. CT = computed tomography, MRI = magnetic resonance imaging.

The patient was administered VPA (1200 mg, intravenously) during the first 2 postoperative days. Because of her stomach and intestine functional recovery, the VPA dosage was changed to 500 mg via oral administration twice a day. A brain MRI was performed on postoperative day 3, and it revealed that the tumor was radically removed and the operative field was clean (Fig. 2A and B). A subsequent paraffin histological examination confirmed the diagnosis of meningioma.

At 3 am on postoperative day 4, the patient was suddenly found in a deep coma. Her pupils were dilated to 3.0 mm and round, and the light reflex was sensitive. The neurological examination was unremarkable, and her vital signs were stable; however, her mental state gradually changed to a deep coma (GCS = 8), which we believed was caused by intracranial lesions. A computerized tomography (CT) scan of the brain was immediately performed; however, it failed to reveal any abnormalities, such as severe brain swelling, intracranial hemorrhage, or cerebral infarction (Fig. 2C). Because a possible cerebral infarction could not be excluded within 24 hours, glucocorticoid, mannitol, and a vasodilator were used. The patient's routine laboratory results for blood cell, liver, and renal functions were in relatively normal ranges. The albumin was 33 g/L, the TBil was 13.3 μmol/L, and the DBil was 4.5 μmol/L. ALT was 12 U/L, and the INR for coagulation values was 1.01. Because of her history of UGH, we checked her blood ammonia levels and performed a fecal occult blood test and blood gas analysis. Her blood ammonia was elevated (181.6 μmol/L; normal range is 11–32 μmol/L), whereas the fecal occult blood test was positive. Thus, considering her history and elevated blood ammonia, she was diagnosed with UGH-induced hepatic encephalopathy, and the oral administration of VPA was changed to intravenous administration to prevent seizures. Lactulose, vitamin B, and L-arginine were administered, clysis with vinegar was performed, and the patient was fasted. The patient's blood ammonia level fluctuated (144.8–207.7 μmol/L), and she remained unconscious during the following 3 days.

Because of the unremarkable progress in the patient's mental state and blood ammonia level, we held a multidisciplinary conference to discuss diagnosis and treatment, initially considering hemodialysis or peritoneal dialysis. After the multidisciplinary discussion, a suspected diagnosis of VHE was finally made, even though the blood VPA level was within the normal range. We decided to stop VPA administration before performing any dialysis and administered levetiracetam instead of VPA. The patient's blood VPA and ammonia levels were then routinely monitored.

**Figure 3:**
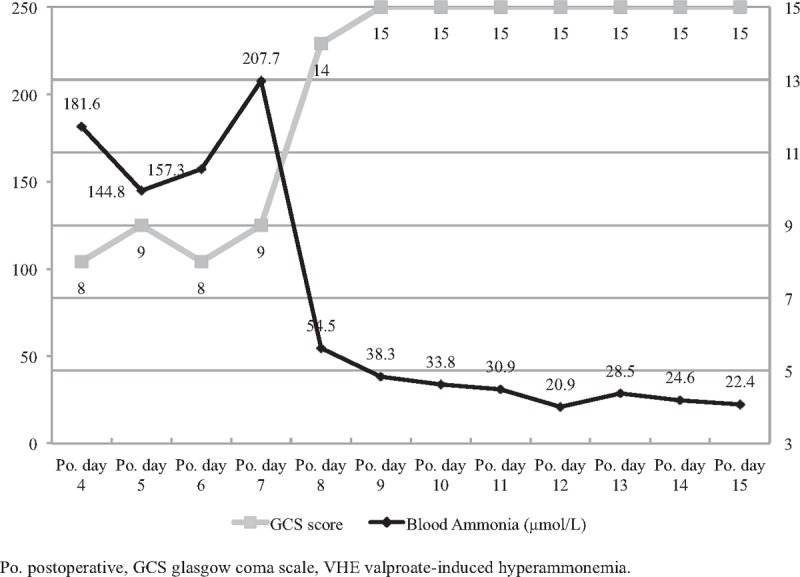
Postoperative blood ammonia and GCS score changes. This figure starts on postoperative day 4 when the patient was suddenly found in a deep coma. On postoperative day 7, discontinuation of VPA began. The patient's mental state improved within 48 hours, and her blood ammonia level returned to normal within 4 days. GCS = Glasgow coma scale, Po. = postoperative, VPA = valproate.

The patient's mental state began to improve from the first 24 hours of VPA discontinuation, and she achieved complete consciousness within 48 hours (GCS = 15). The ammonia levels in her blood decreased dramatically and returned to normal within 4 days (Fig. 3). On postoperative day 15 (8 days after the discontinuation of VPA), the patient was discharged with no complications. The follow-up interviews in postoperative month 5 witnessed her full consciousness and non-recurrence of the primary tumor (Fig. 2D). The patient's GCS score was 15, and she reported enjoying a satisfying quality of postoperative life.

## 3 Discussion

State of consciousness alterations in patients after brain surgery are not uncommon in clinical practice; however, reports of VHE after craniotomy are uncommon.^[3,4,7–9]^ We reviewed the literature published in English and excluded patients who were preoperatively diagnosed with epilepsy and subject to the long-term administration or overdoses of AEDs. We found 6 cases of VHE after craniotomy (Table 1).^[3,4,7,9]^ Our case is unique because of the complicated differential diagnosis of UGH and the neglect of VHE in the early stage. This article should provide a reminder to neurosurgeons to perform a proper differential diagnosis when encountering this sort of patient and initiate effective treatment in a timely manner.

**Table 1:**
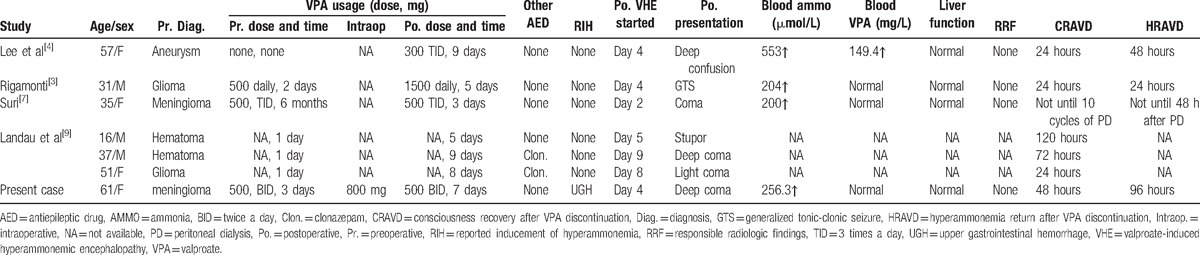
Review of 6 cases published in the literature on VHE after craniotomy.

Postoperative mental state changes in brain tumor patients include coma, unconsciousness, seizures, and tremors. The possible causes of such changes are brain swelling, intracranial hemorrhage, cerebral vasospasm, acute cerebral infarction, electrolyte disturbance, brain-stem injury, seizure, hypoglycemia, and anesthetic effects.^[4,5]^ Searching for the exact cause, such as hyperammonemia as a side effect of VPA, which has long been neglected, represents a challenge in clinical practice.

VPA is recognized as a type of AED and is generally used in neurology and psychiatry for patients diagnosed with epilepsy, dementia, mania, bipolar disorder, social phobias, or neuropathic pain.^[5,6,10]^ Seizures occur in approximately half of patients who have undergone craniotomy without AED administration. Due to an ambiguous recommendation regarding the selection of a prophylactic AED for craniotomy and the excellent capacity of VPA to control perioperative epilepsy, as well as its convenient administration and high tolerance by patients, VPA was used to prevent perioperative seizures in brain tumor patients.

Similar to other medications, VPA has certain recognized side effects, including gastrointestinal disorders, hepatotoxicity, renal toxicity, myelosuppression with blood cell changes, coagulopathy, and irreversible brain injury.^[4,5,11,12]^ Although an elevated level of blood ammonia has been found in as many as half of VPA-treated patients, most of them are asymptomatic.^[3,5,6,13,14]^ VHE is the least common but most serious symptomatic side effect of VPA.^[7]^

Studies concerning the pathogenesis of VPA-induced hyperammonemia and subsequent encephalopathy have been previously reported.^[3,5,15–17]^ VPA inhibits carbamoyl phosphate synthetase I, the key enzyme in the process of converting ammonia into urea in the liver, which leads to a toxic level of blood ammonia. Elevated ammonia can contribute to extracellular glutamate accumulation and Krebs cycle blockages, thereby promoting neurocyte damage and even death. In addition, ammonia is a substrate for more than 16 brain enzymes, and ammonia concentration changes can alter the activities of some of the most important enzymes for brain cell metabolism, including glutaminase, glutamine synthetase, and glutamate dehydrogenase. Such altered activities can contribute to cerebral metabolism disorders and trigger a spectrum of neurological and neuropsychiatric disturbances in clinics.^[18]^

UGH is observed in postoperative brain tumor patients, and it acts as the major cause of elevated blood ammonia and contributes to the development of hepatic encephalopathy with the same pathogenesis as VPA.^[19]^ After the diagnosis of UGH-induced hepatic encephalopathy and the administration of lactulose treatment, including vitamin B, L-arginine and clysis with vinegar, the patient's blood ammonia did not decrease to within normal range but showed fluctuations. However, a rapid decline in ammonia was achieved when VPA was replaced with levetiracetam. Our experience strongly demonstrates that UGH only initiated elevated blood ammonia and that the side effect of VPA, VHE, was the actual cause of postoperative unconsciousness.

Postoperative behavioral and mental changes can be easily misdiagnosed as surgical complications^[4]^ and may lead to the inappropriate decision to maintain or increase the dosage of VPA. Because of the deleterious progression of VHE, including ataxia, coma and death, a differential diagnosis should be highlighted for these patients.

Regarding the risk factors for developing VHE, many conditions are identified, including poor liver function, polypharmacy, intravenous drug administration, carnitine deficiency, and hypercatabolic state.^[4,5]^ Thus, in clinical practice, the above factors should be considered, and enhanced monitoring and adjusted treatment are appreciated in these high-risk patients.

Immediate discontinuation of VPA and performing adjuvant therapy to reduce ammonia, including the administration of lactulose, rifaximin, l-arginine, carnitine, and clysis, are efficient therapies for VHE upon diagnosis.^[3,4,7,11,15]^ Hemodialysis or peritoneal dialysis, which can effectively eliminate blood ammonia and VPA, is the ultimate therapeutic option when ammonia levels do not react to the above therapies.^[7,15,20]^ According to the literature and the present case study, the prognosis for patients with VHE or asymptomatic hyperammonemia is excellent, and the improvements to consciousness correlate well with reduced blood ammonia.

## 4 Conclusions

VHE following craniotomy for brain tumor resection is observed in patients administered perioperative prophylactic VPA. When neurological examinations and emergency radiological findings are negative and an increased dosage of VPA is inefficient, the possibility of VHE should be considered. Postoperative VHE can be definitively diagnosed when unconsciousness and elevated blood ammonia present. In this manner, UGH-induced hepatic encephalopathy should be excluded. Immediate discontinuation of VPA is the most effective method of reducing blood ammonia and restoring consciousness. Administration of VPA should strictly conform to the indications, and the liver function, ammonia blood levels, and VPA dosage of these patients should be monitored.
